# Assessment of knowledge, attitude and practices among dental technicians toward digital laboratory procedures in India: A cross-sectional study

**DOI:** 10.6026/973206300213192

**Published:** 2025-09-30

**Authors:** Surender Kumar, Sandeep Kumar, Madhu Ranjan, Shantala R. Naik, Tushar Sinha, Awanindra Kumar Jha

**Affiliations:** 1 Department of Prosthodontics, Dental Institute, RIMS, Ranchi, Jharkhand, India; 2 Department of Public Health Dentistry, Dental Institute, RIMS, Ranchi, Jharkhand, India; 3 Department of Oral Medicine and Radiology, Dental Institute, RIMS, Ranchi, Jharkhand, India; 4 Department of Prosthodontics, ESIC Dental College & Hospital, Kalaburgi, Karnataka, India; 5 Department of Orthodontics, Dental Institute, RIMS, Ranchi, Jharkhand, India

**Keywords:** Dental laboratory technician, digital dentistry, CAD/CAM, 3D printing

## Abstract

Digitalization in dentistry, encompassing use of digital technology, is indeed the dominant force, impacting every aspect of the profession. Therefore, it is of interest to evaluate and comprehend dental technicians' knowledge, attitude and practice related to digital laboratory procedures in India. A questionnaire-based cross-sectional research was performed among registered dental technicians across India and descriptive statistics were utilized to calculate frequency distributions and chi-square for relationships among variables. The mean knowledge score (4.63±2.18), attitude score (4.52±2.19) and the practice score (4.55±2.14) was substantially exceeded in the dental technicians working in the south zone compared to other zones. The dental technicians employed at private dental laboratory had greater knowledge score (4.04±1.75), attitude score (3.96±1.74) and practice score (3.98±1.72) compared to those working in dental college. The dental technicians of south zone exhibited greater attitude, knowledge and practice of digital dental laboratory procedures than other zones across India.

## Background:

Over the past decade, the accretion of digital technologies has fundamentally revolutionized dental practice. Countless possibilities of digitalization in dentistry have emerged from motivating and educating patients, recording digital impressions to virtual articulators, shade matching, designing and prostheses construction with CAD-CAM (Computer Aided Designing and Computer Aided Manufacturing) technology, Rapid Prototyping etc [1, 2]. Digitalization is becoming the dominant force in dental care, influencing all facets of the profession, from patient treatments to education and lab work [3]. Rapid Prototyping or AM (Additive Manufacturing) is digital process incorporating three-dimensional (3D) printing involves layer-wise assembling of a desired material to fabricate a structure employing CAD/CAM technology [1, 4 and 5]. This process transforms digital data incorporating scanners or CBCT (cone-beam computed tomography) scans into a tangible physical object through a series of steps involving digital modeling, Standard tessellation file (STL) preparation and layer-by-layer fabrication using 3D printer [3]. AM technology is being utilized widely in dentistry from dental model prototypes to splints and surgical guides, prostheses like crowns, bridges, laminates, orthodontic aligners [5, 6, 7-8]. 3D printing performs a crucial task in maxillofacial rehabilitation and Implant dentistry, functional tissues regeneration (hard and soft tissue) with various advantages of rapid processing, high accuracy, customization and fabricating removable to fixed prostheses [4, 9, 10, 11, 12-13]. The absence of worn cutting tools provides high precision and mass production over subtractive techniques [14, 15]. AM can fabricate any article irrespective of its structural intricacy or quantity. It can produce weightless prostheses and also reduces material waste by 40% [4]. 3D printing technology can be categorized into three group; VPP (Vat photopolymerization), that involves DLP (Digital light processing), SLA (Stereolithography); PBF (Powder bed fusion), that mainly incorporates metal materials, comprises SLS (Selective laser sintering), SLM (Selective laser melting), EBM (Electron beam melting) and Fused deposition modeling (FDM) which adopts photosensitive resin materials. All these inherit their pros and cons [5, 14]. The ingress of various digital laboratory technologies in dentistry entails the dental technicians and dental clinicians to be acquainted or updated on advanced knowledge of functionality as well as its application in their daily practice [16]. Dental technicians are essential to the production of highly accurate dental prostheses [17]. Studies conducted in India and abroad revealed that dental technicians possess sound perception about advanced laboratory dental procedures (3D printing, CAD/CAM etc.) and majority showed positive attitude towards implementation of these technologies in their daily workflow. However, in Indian scenario very few studies have been conducted in limited district of Karnataka but presently no data is available pan India. There was a need to conduct a study on a larger scale involving every zone in order to have a better idea regarding the knowledge and awareness of dental technicians towards digital laboratory practices. Therefore, it is of interest to evaluate and comprehend dental technicians' knowledge, attitude and practice related to digital laboratory procedures in India.

## Materials and Methods:

## Study design and setting:

A questionnaire-based cross-sectional research has been carried out among registered dental technicians working in various dental colleges or private dental laboratories from each of the four zones of India (north, south, east and west). The questionnaire was designed on Google Form and the link was circulated to the participants via e-mail and WhatsApp. The study was conducted from February 2025 to April 2025 using convenience sampling method. Criteria of STROBE (Strengthening the reporting of observational studies in Epidemiology) have been followed during research.

## Study population:

Selection criteria involved registered dental technicians (males, females) with above 6 months of experience employed at either dental college or private dental laboratory across India. They should possess basic skills in crown and bridge, orthodontic myo-functional appliance, orthodontic retainers and dental implant prosthesis fabrication. Non-registered and non-practicing dental technicians have been excluded from investigation.

## Study size estimation:

Using proportion of population that was familiar with digital laboratory methods from previously published literature [1], sample size has been calculated using the formula N = [Z2 x P (1 x P)] / e2 at 95% confidence interval and 5% margin of error. After calculating a total sample size of 360 using 10% attrition rates, a final sample size of 400 (100 samples from each zone) was obtained.

## Validation of questionnaire:

One well-structured questionnaire has been drafted with discussion amid senior dental technician working in department of Prosthodontics, Pedodontics and Orthodontics. After conducting pilot study with 10 dental technicians, who were not included in the final analysis, the questionnaire was validated. Five paramedical educators performed the face validity. Ten dental technicians were tested and retested at 2-week intervals to verify instruments reliability (r = 0.82). Questionnaires have been tested for its face, content, criterion and construct validity by two trained experts in the subject. A Pearson correlation analysis was performed and the values of all the items of the questionnaire were checked one by one by the trained specialist. All the questions in which the Pearson correlation value obtained was greater than the critical value and was highly significant were included in the main questionnaire as they were the valid questions. The self-administered questionnaire comprised of 16 closed-ended questions in English language which included demographic data (age, working experience and place of practice), Knowledge (8), Attitude (4) and Practice (4) related questions regarding the digital dental laboratory procedures. The questions were related to 3D printing, CAD-CAM, Guided Implant stents, Orthodontic retainer, night guard appliances etc. The Google Form link was distributed to 400 dental technicians employed at various dental colleges and private dental laboratories across each zone of India. Each participant was explained the purpose and duration of the study in their own language and ensured that their responses was kept anonymous and confidential. If any volunteer denies to participate, the questionnaire page was redirected to the submit section.

## Ethics approval and consent to participate:

This research has been approved by Institutional Ethics Committee of Rajendra Institute of Medical Science, Jharkhand, India (IEC/RIMS17/18.01.2025). Before beginning the survey, Informed consent has been attained from each participant in accordance with recommendations of Declaration of Helsinki.

## Data analysis:

Individual responses were received and transferred to Microsoft Excel sheet. Data has been analyzed utilizing SPSS (Statistical Package for the Social Sciences) Software, v.21.0 (IBM Corp, USA). Descriptive statistics was utilized to calculate frequency distributions and chi-square for relationships among variables. Significance level of has been considered to be <0.05.

## Results:

Survey was distributed among 400 Indian dental technicians. Total completed surveys received were 380(response rate = 95%). There was an equal representation of the dental laboratory technicians from all four zones classified for the study. More than two-third of the respondents were male (88.2%) exhibiting more than five years of work experience (76.6%) in the relevant field. Also, the preponderance of the participants reported to be working in the private dental laboratory (65.8%) (Table 1 - see PDF). Table 2 (see PDF) presents the mean knowledge score (4.63±2.18), attitude score (4.52±2.19) and practice score (4.55±2.14), which was significantly higher in the dental technicians residing in the south zone compared to other zones. As assessed by the post hoc Tukey's test, the dental technicians residing in the north, east and west zones did not display any significant difference in their knowledge, attitude and practice scores. Table 3 (see PDF) presents the mean knowledge score (4.04±1.75), attitude score (3.96±1.74) and practice score (3.98±1.72) which was significantly higher in the dental technicians working in the private dental laboratories compared to those working in the dental colleges. A significant (p value less than 0.001*) strong positive correlation (r value=0.937-0.961) was observed between the mean knowledge, attitude, practice scores (Table 4 - see PDF).

## Discussion:

The process of converting an image into digital code using scanning, tracing, graphics tablet, or analog-to-digital conversion equipment is known as "digitization". It is the process of converting an analog object such as document, artifact, sound, performance or natural phenomenon to a digital copy or recording. Dentistry has advanced from earlier basic treatments such as restorations made of wires and wood to the new era of digital dentistry [4]. Over past couple of decades dentistry has revolutionized adopting CAD/CAM, 3D printing-like technologies for laboratory procedures giving promising results [10]. We had the advantage of conducting the study at a multicentric level across India, using a standardized questionnaire to collect all data, taking an adequate sample size and incorporating homogeneous populations. In the present study there was an equal representation of dental laboratory technicians from each of the four zones suggesting a homogenous population. Majority of the participants had experience greater than five years suggesting that they have worked in their respective field since a long time. Female: Male ratio was 1:7 which shows the need for educating and encouraging females to enroll in the course of lab technicians. Our study revealed that knowledge, attitude and practice scores of dental technicians towards digital laboratory procedures were significantly higher in south zone of India which corroborates to research done by Acharya *et al.* [1] in Karnataka, India. In their study they revealed that awareness related to utilization of digital technology in dentistry has been recognized by 89.53% of technicians. Similar findings have been reported by Klara Loges and Victor Tiberius [14] in their Delphi study performed in Germany. In our study the authors believe that the increased knowledge, attitude and practice scores obtained in the southern zone may be due to higher literacy, higher per capita income and higher demand for advanced prostheses and better utilization of dental services in southern India. An increased number of private dental labs pioneered with digital equipments are established in south, hence giving an opportunity for the working dental technicians to have a greater exposure to digital technology resulting in increased awareness and practice. On contrary, our study reported low knowledge, attitude and practice score in eastern zone. This may be due to low per capita income, fewer established advanced dental clinics, time-consuming advanced dental procedures, lower utilization of digital dental services in eastern India. Another strong reason could be the lack of knowledge regarding the digital laboratory systems in the educational course which corroborates with a study performed by Hsu *et al.* [17] who compared digital practices in dental technicians' education among five Asian countries and revealed that the digital laboratory procedures were deficient in their curricula. The private dental lab technicians had greater digital laboratory knowledge, attitude and practice compared to dental college technicians which were similar to Acharya *et al.* [1] study stating that knowledge and awareness of digital laboratory technology by dental technicians working at private laboratories were greater than those employed at dental colleges. Blackwell *et al.* [18] reported that majority of the UK and Irish dental technicians had knowledge of CAD/CAM technology and were working in large private dental laboratories. Our findings can be attributed to an increased number of dental clinics resulting in increased demand for laboratory services. Moreover, the general population is likely to visit private dental clinics for oral care compared to dental colleges. As a result, private dental labs are more advanced to fulfill the dental needs of the population.

Significant positive correlation has been detected among knowledge score, attitude as well as practice score. As per authors, it can be interpreted that the lab technician who had better knowledge also had better attitude and practice towards digital lab procedures. Therefore, if we enhance the knowledge of dental technicians, their attitude and practice will be improved accordingly. It is recommended that the various training and awareness programs, conferences, etc. of the dental professionals should also include dental technicians to enhance their awareness and practice. Dental technician course providers should adopt hybrid approach including digital learning along with traditional hands-on training in the curriculum to enhance their modern digital practice skills [17]. It is also urged that Dental Council of India take into consideration the need to upgrade the dental technicians post in the dental colleges and incorporate advanced laboratory equipments to update the practice of the faculties as well as the working dental technicians. Considering this, efforts have been initiated by the Indian Prosthodontics Society to train the dental technician along with prosthodontists in various conferences and workshops to upgrade their knowledge and skills.

## Limitations and strengths:

Our research had certain drawbacks that it was a cross-sectional questionnaire-based study with unequal representation of dental technician working in private dental laboratories and dental colleges across India. This study was conducted in an online mode; therefore, chances of bias may have occurred during response collection. Also, those who were not reachable through social media or e-mail were missed out. This study exhibits several strengths as it was conducted in a large geographical region of a developing country. The study was conducted in both educational institutes/colleges and private sectors incorporating a large population in consideration.

## Conclusion:

Data shows that technicians employed in the private laboratories of southern region exhibited greater knowledge, attitude and practice of digital laboratory procedures compared to other regions across India. There is an urgent need to familiarize the dental technicians, especially those employed at dental colleges with successful implementation of digital laboratory technology into dental practices through various training and awareness programs at larger scale. Adapting to new technologies requires upgrading their educational curriculum and promoting national and international collaboration will be essential in fostering career growth of dental technicians, eventually promoting positive oral health.
